# Supplementary material for: Quantum theory based on real numbers can be experimentally falsified
Source: Nature. 2021 Dec 15;600(7890):625–9. doi: 10.1038/s41586-021-04160-4 (PMC8695381; doi:10.1038/s41586-021-04160-4)
Supplement: Supplementary file 1 — This file contains Supplementary Text and Data Sections 1–9. [file 41586_2021_4160_MOESM1_ESM.pdf]

nature portfolio

https://doi.org/10.1038/s41586-021-04160-4

Supplementary information

Quantum theory based on real numbers can be experimentally falsified

In the format provided by the authors and unedited

Quantum theory needs complex numbers
Supplementary Information

Marc-Olivier Renou[1], David Trillo[2], Mirjam Weilenmann[2], Le Phuc Thinh[2], Armin Tavakoli[2,3], Nicolas Gisin[4,5], Antonio Acín[1,6] and Miguel Navascués[2]
[1]ICFO-Institut de Ciencies Fotoniques, The Barcelona Institute of Science and Technology, 08860 Castelldefels (Barcelona), Spain
[2]Institute for Quantum Optics and Quantum Information (IQOQI) Vienna, Austrian Academy of Sciences
[3]Institute for Atomic and Subatomic Physics, Vienna University of Technology, 1020 Vienna, Austria
[4]Group of Applied Physics, University of Geneva, 1211 Geneva, Switzerland
[5]Schaffhausen Institute of Technology – SIT, Geneva, Switzerland
[6]ICREA-Institució Catalana de Recerca i Estudis Avançats, Lluis Companys 23, 08010 Barcelona, Spain

In this Supplementary Information, we formally state and prove the results mentioned in the main text. It is organized as follows. In section I, we discuss the status of postulate (*iv*) in algebraic quantum field theory. In section II, we describe existing real quasi-quantum theories that have the same predictive power as complex quantum theory. In section III, we counter two common theoretical arguments against real quantum theory. In section IV, we show that independently prepared states jointly measured in a single location cannot rule out real quantum theory. In section V we introduce the considered experimental scenario and state our main results, that we prove in sections VI, VII and VIII. In section IX we discuss the challenges and assumptions of a future experimental implementation.

I. TENSOR PRODUCTS IN ALGEBRAIC QUANTUM FIELD THEORY

Postulate (iv) is generally believed not to apply in quantum field theory, where space-like separation is usually modeled through commutation relations rather than tensor products. In this section, we show that, contrary to this widely-held opinion, postulate (iv) is a necessary condition for quantum field theories to be *physical*.

Algebraic quantum field theory (AQFT) is an axiomatization of quantum field theory developed by Haag, Kastler, Araki and others in the 1950s [1]. The starting point of AQFT is a space-time manifold M and an overall Hilbert space $\mathscr{H}$. For any space-time bounded region $O \subset M$, there exists a C^*-algebra $A(O)$, acting on $\mathscr{H}$, whose elements correspond to the set of measurements and operations that an experimenter acting in O can conduct. Several postulates try to capture the properties of these algebras if they are to represent the local observables of a reasonable quantum field theory. We will just mention one: If $O_1, O_2 \subset M$ are causally unconnected, then $[A(O_1), A(O_2)] = 0$. This condition, called microcausality, formalizes the notion that experimenters acting in causally unconnected regions can conduct operations independently of one another.

In a quantum field theory we do not only expect to be able to conduct independent measurements, but also independent local state preparations. In the language of AQFT, a local state ϕ in the region $O \subset M$ corresponds to a linear functional $\phi : A(O) \to \mathbb{C}$ such that $\phi(xx^\dagger) \geq 0$, for all $x \in A(O)$, and $\phi(1) = 1$. The state is called *normal* if there exists a trace-class positive semidefinite operator ρ acting on $\mathscr{H}$ such that $\phi(x) = \mathrm{tr}\rho x$ for all $x \in A(O)$. Intuitively, in order to prepare a local state in a region O, we might need to act on a slightly larger region O' (e.g.: to shield O from cosmic rays). Denoting by $B(\mathscr{H})$ the set of bounded operators acting on $\mathscr{H}$, a local preparation of ϕ is thus defined as a completely positive map $T : B(\mathscr{H}) \to B(\mathscr{H})$ such that $T(x) = \phi(x)T(1)$, for all $x \in A(O)$ and $T(y') = T(1)y'$, for all y' commuting with $A(O')$, where $O' \in M$ represents a space-time region that strictly contains O. Note that, in principle, $T(1) \neq 1$, i.e., we allow for the preparation to be non-deterministic.

As shown by Werner [2], the possibility of conducting, for any bounded $O \in M$, a non-deterministic local preparation of some normal state ϕ implies that one can prepare any normal state in O deterministically by means of maps T of the form $T(\omega) = \sum_j c_j \omega c_j^\dagger$, with $c_j \in A(O')$. In [2], this condition is also proven equivalent to the so-called *split property*. The split property demands, for every $O, O' \subset M$ with O strictly contained in O', the existence of a type-I von Neumann factor (namely, a C^*-algebra isomorphic to $B(\mathscr{K})$ for some Hilbert space $\mathscr{K}$) A such that $A(O) \subset A \subset A(O')$. Due to the previous considerations and the fact that large classes of solvable models satisfy it [3, 4], the split property is usually regarded as an extra postulate of AQFT, when it is not derived from general assumptions of thermodynamic stability [5].

How does the split property relate to experiments with space-like separated experimenters? Type-I factors A have the convenient property of factoring the Hilbert space $\mathscr{H}$ where they act. Namely, if A is a type-I factor, then there exist Hilbert spaces $\mathscr{K}$, $\mathscr{K}'$ and a unitary $U : \mathscr{H} \to \mathscr{K} \otimes \mathscr{K}'$ such that $UAU^\dagger = B(\mathscr{K}) \otimes \mathbb{I}_{K'}$ and $UA'U^\dagger = \mathbb{I}_K \otimes B(\mathscr{K}')$, where the commutant A' is the set of operators in $B(\mathscr{H})$ that commute with all the elements of A.

Now, consider a scenario where two parties Alice and Bob conduct measurements in a space-like separated way. Call O_A (O_B) the space-time region where Alice (Bob) conducts her (his) experiments, and suppose that there exists a region O'_A that strictly contains O_A and such that O'_A, O_B are space-like separated. Then, by virtue of the split property, there exists a type-I algebra A such that $A(O_A) \subset A \subset A(O'_A)$. Since by microcausality $A(O_B) \subset A(O'_A)' \subset A'$, it follows that there exists a unitary $U : \mathscr{H} \to \mathscr{K} \otimes \mathscr{K}'$ such that $UA(O_A)U^\dagger = \tilde{O}_A \otimes \mathbb{I}_{\mathscr{K}'}$, $UA(O_B)U^\dagger = \mathbb{I}_{\mathscr{K}} \otimes \tilde{O}_B$. That is, the interactions of each party can be understood to take place in different factors of an overall Hilbert space. This argument generalizes for arbitrarily many parties.

In multi-partite scenarios in AQFT, the overall Hilbert space can thus be decomposed as the tensor product of multiple factors, one for each party, and the physical operations carried out by each party are represented by operators acting trivially on all the other factors. In this framework, if each party conducts a local state preparation (as defined above), the statistics observed by all the parties can be seen to correspond to those generated by a product quantum state. Hence postulate (iv) in the main text also holds in quantum field theories satisfying the split property.

II. REPRODUCING THE PREDICTIONS OF QUANTUM MECHANICS THROUGH NON-LOCAL REAL THEORIES

Following the development of quantum theory, alternative theories that retain only part of the quantum formalism were developed, usually with the aim to enforce certain desirable properties.

In his 1960 seminal paper [6], Stueckelberg tackled the problem of constructing a version of quantum theory that does not require complex numbers. His construction is mathematically equivalent to the one that Regina proposes in the main text to explain Conan's single-site experiments. In his work [6], Stueckelberg is just concerned with single-site experiments, so he does not provide a prescription to describe multipartite experiments.

A straightforward generalization of Stueckelberg's theory, advocated in [7], consists in positing the existence of a universal quantum bit, partially accessible to each observer. In this theory, the Hilbert space $\mathscr{H}$ of any quantum system factors into as many Hilbert spaces as independent subsystems, plus the universal qubit U. The operations that an experimenter with access to factor A can perform are of the form

$$|i\rangle\langle i|_U \otimes O_A \otimes \mathbb{I}_{\tilde{A}} + |-i\rangle\langle -i|_U \otimes O_A^* \otimes \mathbb{I}_{\tilde{A}}, \tag{1}$$

where $\tilde{A}$ denotes all factors decomposing $\mathscr{H}$ other than A and U. This generalization recovers all quantum predictions. If one identifies the basic elements of this theory (namely, vectors and projectors) with those of quantum theory, then one finds that Stueckelberg's theory violates postulate (iv) in the main text, as all subsystems are allowed to operate on factor U. One can thus interpret Stueckelberg's theory as a non-local (yet non-signalling) real variant of quantum theory.

Another alternative that deserves specific mention, for its similarities to Stueckelberg's theory, is Bohmian mechanics [8]. Here, in addition to the quantum wave function, particle positions are part of the ontology. Even though it is generally formulated over complex Hilbert spaces, Bohmian mechanics can incorporate the above construction using a universal qubit. Violating postulate (iv) may be considered a less serious flaw in this case, as the wave function is not associated with the states of individual particles but rather guides their dynamics in a fundamentally non-local way anyway. No wonder, this theory is usually regarded as a non-local (yet non-signalling) classical theory.

III. NON-EXPERIMENTAL ARGUMENTS AGAINST REAL QUANTUM THEORY

Our work proves that real quantum theory, i.e., the theory satisfying the postulates $(i_{\mathbb{R}})$, $(ii)-(iv)$, cannot reproduce the predictions of complex quantum theory in the entanglement swapping scenario. Prior to our work, some arguments against real quantum theory, also called real quantum mechanics [9, 10], have appeared in the quantum foundations literature. As we explain below, such arguments are not associated to any experimental observation: rather, they amount to non-falsifiable conceptual considerations related to the internal properties of the theory.

A natural argument against a real quantum theory is based on dimension considerations. For instance, photon polarization is described by a complex Hilbert space of dimension 2, hence is determined by three independent parameters n_x, n_y, n_z. In contrast, if the Hilbert space of photon polarization were real, then just two numbers n_x, n_z suffice to describe a physical state. The successful encoding and decoding of three parameters in identical preparations of a photon state seems to be a solid argument against real quantum theory.

Note, however, that Hilbert space dimension cannot be experimentally lower bounded. Interpreting such an experiment as a disproof of real quantum theory thus requires a considerable leap of faith. In fact, real quantum theory

can account for the measurement statistics of any such experiment by postulating that photon polarization lives in a 4-dimensional real Hilbert space. According to such a real quantum interpretation of photon experiments, photon polarization would allow storing, not just three, but nine continuous parameters (as such is the number of independent components of a real 4×4 normalized density matrix). The fact that we do not seem to have access to those six extra degrees of freedom can be explained away by appealing to some super-selection rules, or more simply to our own limitations as experimental physicists.

A second argument often heard against real quantum theory is the fact that it violates the principle of local tomography: namely, there exist distinct multiparty real quantum states which cannot be distinguished via local measurements. Local tomography has been introduced in several axiomatizations of quantum mechanics, see e.g. [11, 12]. However, whether this property should be regarded as a reasonable physical principle is controversial, as evidenced by the interest in physical models that clearly violate it [9, 10, 13]. Note also that, like the dimensional arguments, any observed violation of local tomography can be explained away by arguing that the local measurements conducted on each subsystem were not exhaustive.

Other abstract mathematical arguments against (or in favor of) real quantum theory exists, based on symmetry considerations [14–16]. None are experimentally falsifiable.

IV. REAL SIMULATION OF JOINT MEASUREMENTS ON INDEPENDENT PREPARATIONS

There exist no-go theorems in quantum physics, such as the Pusey-Barrett-Rudolph (PBR) theorem [17], that deal with a scenario in which N independent sources prepare quantum states that are sent to a central node, where joint measurements are performed. Our goal here is to provide a strategy for the simulation using real quantum theory of any such experiment in complex quantum theory. In what follows, we restrict the analysis to the case in which each source prepares P possible states and only one measurement is performed, the generalization to more measurements being straightforward.

In the considered scenario, each source i, with $i = 1, \ldots, N$, prepares the complex states $\{\rho^{(i)}_{p_i}\}_{p_i=1,\ldots,P}$, which are sent to a central node that performs on the N states the measurement of R possible results defined by the complex operators $\{M_r\}_{r=1,\ldots,R}$. The obtained statistics is described by the conditional probability distribution

$$P(r|p_1 \ldots p_N) = \mathrm{tr}\Big(\rho^{(1)}_{p_1} \otimes \rho^{(2)}_{p_2} \otimes \ldots \otimes \rho^{(N)}_{p_N} M_r\Big). \tag{2}$$

A possible real simulation works as follows. The preparations by each source i are just encoded in a basis of a real Hilbert space of dimension P, $\{|p_i\rangle\}_{p_i=1,\ldots,P}$. The measurement operators are defined by the positive operators

$$M_r = \sum_{p_1,\ldots,p_N} P(r|p_1 \ldots p_N)|p_1 \ldots p_N\rangle\langle p_1 \ldots p_N|, \tag{3}$$

which sum up to the identity. It is simple to see that

$$P(r|p_1 \ldots p_N) = \mathrm{tr}(|p_1 \ldots p_N\rangle\langle p_1 \ldots p_N|M_r). \tag{4}$$

Note that this construction provides an alternative real simulation of any experiment involving a single quantum system different from the one presented in Figure 1 in the main text. We were unable to adapt the construction with the extra qubit in this Figure to the independent preparation scenario considered here. The impossibility comes from the fact that at the measuring device complex conjugation, a positive but not completely positive map, needs to be applied to parts of the system. In fact, based on numerical simulations, we conjecture that there are experiments involving joint measurements on independent preparations in complex Hilbert spaces of dimension d that require for its real simulation a Hilbert space dimension strictly larger than $2d$. On the other hand, the simple encoding of the preparations and measurements in a real basis discussed here does not work in experiments displaying a Bell inequality violation, as they require entanglement, hence quantum coherence. While one should never forget that these are just possible real simulations, not necessarily unique, the conflict appearing between the two approaches gives another intuitive explanation of why a real simulation becomes impossible when combining independent preparations and measurements.

V. OUR SETUP AND MAIN RESULTS

Causal networks are the natural generalizations of Bell scenarios to a richer class of causality relations. In this work, we consider the causal network depicted in Fig. 2 of the main text (lower pane), which is similar to an entanglement

swapping setup and therefore called the *SWAP scenario*. A (shared randomness) source λ controls two spacelike-separated sources S_L, S_R (for left and right source) each of which further generates a bipartite system to be sent towards three distant observers Alice, Bob and Charlie. The observers can choose measurement x, y, z to conduct on the incoming systems locally, and receive an outcome a, b, c. Repetition of the experiment allows them to collect a family of joint probability distributions $P(a, b, c|x, y, z)$. Going forward, in our SWAP scenario, Alice and Charlie have, respectively, three, and six dichotomic measurement settings, i.e., $x = 1, 2, 3; z = 1, ..., 6$ and $a, c = -1, 1$, while Bob's only measurement is assumed to have four outcomes $b = b_1b_2 = 00, 01, 10, 11$, that we equivalently label as $b = \phi^+, \psi^+, \phi^-, \psi^-$ for reasons that will soon be obvious.

Just like in Bell scenarios, the source λ is assumed to be a *classical* source modelled by a classical probability distribution $P(\lambda)$. Moreover, the causal network forbids the possibility that λ influences x, y, z (also known as *measurement dependence*). The reason for classicality is that S_L, S_R may be produced in the same factory or being operated using the same power socket, which are believed to be sources of classical correlations. Furthermore, the causal network with an additional source λ is clearly more general than the one without such source, and in fact our results even hold in this setting.

Interpretation within complex complex quantum theory implies that $P(a, b, c|x, z)$ must be of the form

$$P(a, b, c|x, z) = \sum_\lambda P(\lambda)\text{tr}\left\{(\tilde{\sigma}^\lambda_{AB_1} \otimes \tilde{\sigma}^\lambda_{B_2C})(\tilde{A}_{a|x} \otimes \tilde{B}_b \otimes \tilde{C}_{c|z})\right\}, \tag{5}$$

for some quantum states $\tilde{\sigma}^\lambda_{AB_1}, \tilde{\sigma}^\lambda_{B_2C}$, some probability distribution $P(\lambda)$ and projective measurement operators $\tilde{A}_{a|x}, \tilde{B}_b, \tilde{C}_{c|z}$, with $\sum_a \tilde{A}_{a|x} = \mathbb{I}_A, \sum_b \tilde{B}_b = \mathbb{I}_B, \sum_c \tilde{C}_{c|z} = \mathbb{I}_C$. Interpreting the SWAP scenario according to *real* quantum theory gives exactly the same equation but with all operators restricted to acting on a *real* Hilbert space or, equivalently, having real matrix entries.

Note that, rather than projective measurements, Alice, Bob and Charlie could in principle conduct the more general Positive Operator valued Measures (POVMs), whereby the operators $\tilde{A}_{a|x}, \tilde{B}_b, \tilde{C}_{c|z}$ are just required to be positive semidefinite instead of projectors. However, any correlation $P(a, b, c|x, z)$ admitting a decomposition of the form (5), with $\tilde{A}_{a|x}, \tilde{B}_b, \tilde{C}_{c|z}$ denoting (real) POVM elements, can also be reproduced with (real) projective measurements. This is so because, through Naimark dilations, one express any local (real) POVM as a local (real) projective measurement acting over the original quantum system and a local ancillary system in a (real) pure state. Hence, in order to disprove that a given distribution $P(a, b, c|x, z)$ admits a real quantum representation (5), it suffices to do so under the assumption of projective measurements.

We now describe the specific correlation $\bar{P}(a, b, c|x, z)$ and Bell inequality $\mathscr{T}$ that witness the separation between real and complex quantum theory. Let the two quantum sources distribute the states $\bar{\sigma}_{AB_1} = \bar{\sigma}_{B_2C} = \Phi^+ = |\phi^+\rangle\langle\phi^+|$, where $|\phi^+\rangle = \frac{1}{\sqrt{2}}(|00\rangle + |11\rangle)$. Alice's three dichotomic observables $(\bar{A}_{1|x} - \bar{A}_{-1|x} : x = 1, 2, 3)$ correspond to the three Pauli measurements $\sigma_Z, \sigma_X, \sigma_Y$, and Charlie measures the dichotomic operators

$$\bar{D}_{ij} = \frac{\sigma_i + \sigma_j}{\sqrt{2}}, \qquad \bar{E}_{ij} = \frac{\sigma_i - \sigma_j}{\sqrt{2}}, \tag{6}$$

for $ij = zx, zy, xy$, which correspond to the observables $(\bar{C}_{1|z} - \bar{C}_{-1|z} : z = 1, \dots, 6)$ when taken in the order D_{zx}, E_{zx}, D_{zy}, E_{zy}, D_{xy}, E_{xy}. Bob conducts a Bell basis measurement, with outcomes b corresponding to each of the orthogonal projections onto the states $|\phi^\pm\rangle = \frac{1}{\sqrt{2}}(|00\rangle \pm |11\rangle)$, $|\psi^\pm\rangle = \frac{1}{\sqrt{2}}(|10\rangle \pm |01\rangle)$. We warn the reader that our convention of $|\psi^-\rangle$ differs from the usual one so as to make our formulas simpler.

These define the resulting distribution $\bar{P} = \{\bar{P}(a, b, c|x, z) : a, b, c, x, z\}$, which obviously admits a representation of the form (5), with *complex* measurement operators. Since $\bar{P}$ does not require shared randomness λ for its realization, it is also compatible with the less general causal network depicted in Figure 2 of the main text (upper pane) in the main text.

Given some distribution $P(a, b, c|x, z)$, define $S^b_{xz} = \sum_{a,c=-1,1} P(a, b, c|x, z)ac$ and the linear functional

$$\begin{aligned}\mathscr{T}_b(P) =& (-1)^{b_2}(S^b_{11} + S^b_{12}) + (-1)^{b_1}(S^b_{21} - S^b_{22}) + \\ & (-1)^{b_2}(S^b_{13} + S^b_{14}) - (-1)^{b_1+b_2}(S^b_{33} - S^b_{34}) + \\ & (-1)^{b_1}(S^b_{25} + S^b_{26}) - (-1)^{b_1+b_2}(S^b_{35} - S^b_{36}).\end{aligned} \tag{7}$$

Note that S^b_{xz} is the same as the conditional expectation value $\langle A_x C_z\rangle$ when conditioned on the event that Bob receives the outcome b. Therefore, for $b = 00$ then $\mathscr{T}_{00}(P)$ coincides with the LHS of the CHSH$_3$ Bell inequality for Alice and Charlie, as defined in eq. (2) in the main text, but evaluated for $P(a, b, c|x, z)$; for the other b it corresponds

to variations of the same inequality. It can be verified that, for the considered states and measurements, the identity $\mathscr{T}_b(\bar{P}) = 6\sqrt{2}\bar{P}(b)$ holds for all b, with $\bar{P}(b) = \frac{1}{4}$. This means that, conditioned on any measurement outcome b of Bob's, the state of Alice and Charlie allows them to maximally violate one of the variants of the CHSH$_3$ Bell inequality, namely the one corresponding to $\mathscr{T}_b$.

Our first result, proven in Section VI, is the impossibility to reproduce $\bar{P}$ exactly using real quantum physics.

Proposition 1. *$\bar{P}$ does not admit a decomposition of the form (5) if we demand the states $\tilde{\sigma}^\lambda_{AB_1}, \tilde{\sigma}^\lambda_{B_2C}$ and measurement operators $\tilde{A}_{a|x}, \tilde{B}_b, \tilde{C}_{c|z}$ to be real, regardless of the dimension of the underlying real Hilbert space.*

A more elaborate argument, presented in section VII, leads to the following robustness claim.

Theorem 2. *Let $P(a, b, c|x, z)$ be a distribution such that $|P(b) - \frac{1}{4}| < \varepsilon_c$ and $\mathscr{T}_b(P) > (6\sqrt{2} - \varepsilon_c)P(b)$, for all b, with $\varepsilon_c \approx 7.18 \cdot 10^{-5}$. Then, $P(a, b, c|x, z)$ does not admit a decomposition of the form (5), with real states and measurement operators, regardless of the dimension of the underlying real Hilbert space.*

Maximally violating the Bell inequality CHSH$_3$ with a precision of order ε_c right after a Bell measurement is way beyond the capabilities of current quantum technologies. Define $\mathscr{T}(P) = \sum_{b\in\{0,1\}^2} \mathscr{T}_b(P)$, and note that in the considered setup, $\mathscr{T}(\bar{P}) = 6\sqrt{2} \approx 8.4852$. In section VIII we use ideas from non-commutative polynomial optimization [18] to obtain the experimentally friendly robustness bound.

Theorem 3. *For any distribution P admitting a decomposition of the form (5) with real quantum states and real measurement operators acting on real Hilbert space of arbitrary dimension,*

$$\mathscr{T}(P) = \sum_{b\in\{0,1\}^2} \mathscr{T}_b(P) \leq 7.6605. \tag{8}$$

The figure appearing in the theorem is the solution of a semidefinite program (SDP) [19], a type of convex optimization problem that can be solved in polynomial time. To arrive at this result, we used the SDP solver Mosek [20], with the MATLAB package YALMIP [21]. Note that, from duality theory, we can *certify* that the solution of the SDP is the one stated in the theorem, up to computer precision. That is, the Theorem above represents a rigorous mathematical result, and can be understood as a computer-generated proof.

VI. PROOF OF PROPOSITION 1

We prove the result by contradiction. Suppose that, indeed, there exist a distribution $P(\lambda)$, Hilbert spaces A, B_1, B_2, C, real states $\tilde{\sigma}^\lambda_{AB_1}, \tilde{\sigma}^\lambda_{B_2C}$ and real measurement operators $\tilde{A}_{a|x}, \tilde{B}_b, \tilde{C}_{c|z}$ such that eq. (5) holds with $P(a, b, c|x, z) = \bar{P}(a, b, c|x, z)$. Let $\psi := \sum_\lambda P(\lambda)\tilde{\sigma}^\lambda_{AB_1} \otimes \tilde{\sigma}^\lambda_{B_2C}$ be the global state for systems A, B_1, B_2, C at the beginning of the experiment. With probability $P(b) = \mathrm{tr}(\psi\tilde{B}_b)$, Bob observes outcome b and collapses the AC system into the state $\mathrm{tr}_B(\tilde{B}_b\psi\tilde{B}_b)/P(b)$.

Going forward, consider a real purification $|\psi^b\rangle_{ACP}$ of Alice and Charlie's conditional state, and redefine Charlie's system C to accommodate the purifying system P, over which Charlie's measurement operators act trivially. This simplifies our notation by replacing density matrices on AC by pure states on $A(CP)$. Most importantly, the choice of purification does not impact our result: this is so because the only role that $|\psi^b\rangle_{ACP}$ plays in the proof consists in being acted on system A and the original system C with linear maps, after which the purifying system P is traced out, together with systems A, C. Hence, assuming the existence of such a purification does not affect the final expressions and hence the validity of the proof. Consequently, by a slight abuse of notation, we will from now on refer to the system CP simply as C.

We denote Alice's three dichotomic observables by Z^A, X^A, Y^A, referring to $(\tilde{A}_{1|x} - \tilde{A}_{-1|x} : x = 1, 2, 3)$. Analogously, Charlie's dichotomic observables $(\tilde{C}_{1|z} - \tilde{C}_{-1|z} : z = 1, ..., 6)$ are denoted by $D^C_{zx}, E^C_{zx}, D^C_{zy}, E^C_{zy}, D^C_{xy}, E^C_{xy}$, respectively. We mostly work with the operator version of (7), which in this notation takes the form

$$\begin{aligned}\hat{\mathscr{T}}_b =&(-1)^{b_2}Z^A(D^C_{zx} + E^C_{zx}) + (-1)^{b_1}X^A(D^C_{zx} - E^C_{zx})\\ &(-1)^{b_2}Z^A(D^C_{zy} + E^C_{zy}) - (-1)^{b_1+b_2}Y^A(D^C_{zy} - E^C_{zy})\\ &(-1)^{b_1}X^A(D^C_{xy} + E^C_{xy}) - (-1)^{b_1+b_2}Y^A(D^C_{xy} - E^C_{xy}).\end{aligned} \tag{9}$$

The fact that we start with real projective measurements translates into these nine operators satisfying $O^2 = \mathbb{I}$ and $O^T = O$.

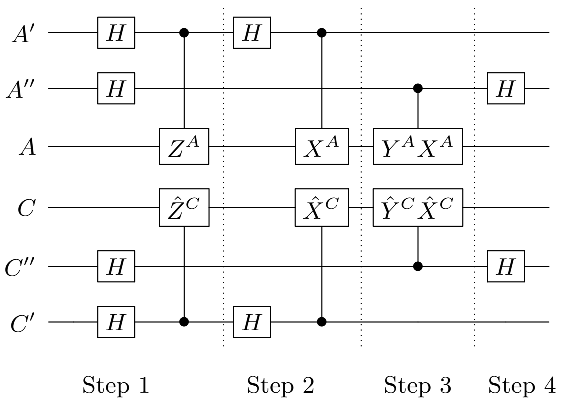

FIG. 1. Real local isometry $U \otimes V$ built from each party's untrusted measurement operators. For later reference, we denote the local isometry performing Steps 1 and 2 of the circuit as $U' \otimes V'$, and the remaining gates (performing the operations of Steps 3 and 4) as $U'' \otimes V''$. The operators $\hat{X}^C$, $\hat{Y}^C$, $\hat{Z}^C$ are defined in terms of Charlie's measurement operators as the regularised versions of $X^C = \frac{D^C_{zx} - E^C_{zx}}{\sqrt{2}}$, $Y^C = \frac{D^C_{zy} - E^C_{zy}}{\sqrt{2}}$ and $Z^C = \frac{D^C_{zx} + E^C_{zx}}{\sqrt{2}}$ respectively. For any operator O let $\hat{O} := O_{0\to 1}|O_{0\to 1}|^{-1}$ where $O_{0\to 1}$ is the resulting operator after setting any zero eigenvalues of O to one. This regularization procedure [22] turns a hermitian operator O into a unitary operator with eigenvalues ± 1. Finally, H denotes the Hadamard gate, defined through the relations $H|0\rangle = \frac{1}{\sqrt{2}}(|0\rangle + |1\rangle)$, $H|1\rangle = \frac{1}{\sqrt{2}}(|0\rangle - |1\rangle)$.

Taking inspiration from [22], we consider the real isometry depicted in Figure 1. Our first claim is that, if the real state $|\psi^b\rangle$ saturates the quantum bound $\langle\psi^b|\hat{\mathscr{T}}_b|\psi^b\rangle \leq 6\sqrt{2}$ for $b = \phi^+, \psi^+, \phi^-, \psi^-$, then the effect of this isometry on systems $A'A''C'C''$ is to prepare the states

$$\rho^b := \mathrm{tr}_{AC}(U \otimes V|\psi^b\rangle\langle\psi^b|U^\dagger \otimes V^\dagger) = |b\rangle\langle b|_{A'C'} \otimes \left[\frac{\Psi^+ + \Phi^-}{2}\right]_{A''C''}. \tag{10}$$

Note that, as promised, the isometry only acts on system A and the original system C (since it is built from Charlie's measurement operators). Systems A and CP are traced out; hence the same final state ρ^b would have been obtained had we not purified Alice and Charlie's post-selected state. Now we prove this equation for the case $b = \phi^+$ only, noting that the other cases are completely analogous (or can be checked in Appendix VII). We explicitly show the effect of the isometry step by step.

- After steps 1 and 2 of the isometry we are left with the state

$$|\psi^{\phi^+}\rangle_{AC}|0000\rangle_{A'A''C'C''} \mapsto |\phi^+\rangle_{A'C'}|++\rangle_{A''C''}\frac{\mathbb{I} + Z^A}{\sqrt{2}}|\psi^{\phi^+}\rangle_{AC}.$$

This follows from eq. (A12) in [22], in turn a consequence of the maximal violation of the CHSH inequality through Alice and Charlie's first two measurement settings. We can now forget about systems $A'C'$, since the subsequent operations defining the isometry do not act on them.

- After Step 3, systems $AA''CC''$ are in state

$$\frac{1}{2}(|00\rangle_{A''C''}|\varphi\rangle_{AC} + |11\rangle_{A''C''}Y^AX^A\hat{Y}^C\hat{X}^C|\varphi\rangle_{AC} + |01\rangle_{A''C''}\hat{Y}^C\hat{X}^C|\varphi\rangle_{AC} + |10\rangle_{A''C''}Y^AX^A|\varphi\rangle_{AC}), \tag{11}$$

where $|\varphi\rangle_{AC} = \frac{\mathbb{I}+Z^A}{\sqrt{2}}|\psi^{\phi^+}\rangle_{AC}$. Since $|\psi^{\phi^+}\rangle$ maximally violates a CHSH$_3$ inequality, the identities

$$\begin{aligned}
&\{X^A, Y^A\}_+|\psi^{\phi^+}\rangle_{AC} = \{X^A, Z^A\}_+|\psi^{\phi^+}\rangle_{AC} = \{Z^A, Y^A\}_+|\psi^{\phi^+}\rangle_{AC} = 0,\\
&\hat{Z}^C|\psi^{\phi^+}\rangle_{AC} = Z^A|\psi^{\phi^+}\rangle_{AC},\\
&\hat{Y}^C|\psi^{\phi^+}\rangle_{AC} = -Y^A|\psi^{\phi^+}\rangle_{AC},\\
&\hat{X}^C|\psi^{\phi^+}\rangle_{AC} = X^A|\psi^{\phi^+}\rangle_{AC}
\end{aligned} \tag{12}$$

hold, see [22] or Appendix F for a detailed proof. In turn, those imply the relations $\hat{Y}^C\hat{X}^C|\varphi\rangle_{AC} = Y^AX^A|\varphi\rangle_{AC}$ and $Y^AX^A\hat{Y}^C\hat{X}^C|\varphi\rangle_{AC} = -|\varphi\rangle_{AC}$. Therefore (11) is the same as

$$\frac{1}{\sqrt{2}}(|\phi^-\rangle_{A''C''}|\varphi\rangle_{AC} + |\psi^+\rangle_{A''C''}Y^AX^A|\varphi\rangle_{AC}).$$

- The Hadamard gates of step 4 change $|\phi^-\rangle_{A''C''}$ to $|\psi^+\rangle_{A''C''}$ and viceversa. The final state is therefore

$$\frac{1}{\sqrt{2}}|\phi^+\rangle_{A'C'}(|\psi^+\rangle_{A''C''}|\varphi\rangle_{AC} + |\phi^-\rangle_{A''C''}Y^AX^A|\varphi\rangle_{AC}).$$

Finally, we take the partial trace over systems AC to obtain

$$\frac{1}{2}\Phi^+ \otimes \left(\langle\varphi|Y^AX^A|\varphi\rangle(|\phi^-\rangle\langle\psi^+| + |\psi^+\rangle\langle\phi^-|) + \langle\varphi|\varphi\rangle(\Psi^+ + \Phi^-)\right).$$

Since $U \otimes V$ is an isometry, it immediately follows that $\langle\varphi|\varphi\rangle = 1$, which is equivalent to saying that $\langle\psi^{\phi^+}|Z^A|\psi^{\phi^+}\rangle = 0$. So far, all our considerations are general and did not use the fact that we are dealing with real numbers. Taking this fact into account, we have that

$$\langle\varphi|Y^AX^A|\varphi\rangle = \overline{\langle\varphi|Y^AX^A|\varphi\rangle} = \langle\varphi|X^AY^A|\varphi\rangle = -\langle\varphi|Y^AX^A|\varphi\rangle.$$

Therefore, this quantity is zero and the state left in systems $A'C'A''C''$ is indeed ρ^b.

To reach a contradiction, note that

$$\frac{\Psi^+ + \Phi^-}{2} = \frac{|i\rangle\langle i|^{\otimes 2} + |-i\rangle\langle -i|^{\otimes 2}}{2} \tag{13}$$

and therefore summing over Bob's results, the application of $U \otimes V$ leaves system $A'A''C'C''$ in the state

$$\rho = \sum_b \bar{P}(b)\rho^b = \frac{1}{4}\left(\rho^{\phi^+} + \rho^{\phi^-} + \rho^{\psi^+} + \rho^{\psi^-}\right) = \frac{\mathbb{I}_{A'C'}}{4} \otimes \left[\frac{|i\rangle\langle i|^{\otimes 2} + |-i\rangle\langle -i|^{\otimes 2}}{2}\right]_{A''C''}. \tag{14}$$

On the other hand, from (5) we have that the same application of $U \otimes V$ should give the state

$$\rho = \sum_\lambda P(\lambda)\mathrm{tr}_A(U\tilde{\sigma}_A^\lambda U^\dagger) \otimes \mathrm{tr}_C(V\tilde{\sigma}_C^\lambda V^\dagger). \tag{15}$$

The last two equations contradict each other. The last state is always a real separable state with respect to the partition $A'A''$ vs $C'C''$ because $\sigma_A^\lambda, \sigma_C^\lambda$ are real quantum states and U, V are real quantum operations. The penultimate state, however, is not real separable (but is complex separable) because it violates a necessary condition for real separability [23]: indeed, the $A''C''$ marginal state is not invariant under the partial transposition of either subsystem. This completes the proof.

VII. PROOF OF THEOREM 2

First, we need to solve the optimization problem

$$\min_{\tau\in\mathscr{S}} \|\tau - \rho_0\|_1, \tag{16}$$

where $\mathscr{S}$ is the set of states in $A'A''C'C''$ invariant under transposition of $A'A''$ and $\rho_0 = \rho$, as defined by the right-hand side of eq. (14).

Let $S_{A'A''}$ be the quantum channel defined by $S_{A'A''}(\bullet) = \sum_{\alpha',\alpha''=\pm i} |\alpha'\rangle\langle\alpha'| \otimes |\alpha''\rangle\langle\alpha''| \bullet |\alpha'\rangle\langle\alpha'| \otimes |\alpha''\rangle\langle\alpha''|$. Note that $S^2_{A'A''} = S_{A'A''}$, that $S_{A'A''} \otimes \mathbb{I}_{C'C''}(\rho_0) = \rho_0$ and that $S_{A'A''} \otimes \mathbb{I}_{C'C''}(\mathscr{S}) \subset \mathscr{S}$. By the monotonicity of the trace norm, we thus have that τ in (16) can be chosen invariant under $S_{A'A''} \otimes \mathbb{I}_{C'C''}$.

Any operator O invariant under $S_{A'A''} \otimes \mathbb{I}_{C'C''}$ is of the form

$$O = \sum_{\alpha',\alpha''=\pm i} |\alpha'\rangle\langle\alpha'|_{A'} \otimes |\alpha''\rangle\langle\alpha''|_{A''} \otimes O^{\alpha',\alpha''}_{C'C''}. \tag{17}$$

It can be verified that the partial transposition of systems A', A'' of any such operator effects the transformation $O \to (\sigma_z^{\otimes 2} \otimes \mathbb{I}^{\otimes 2}) O (\sigma_z^{\otimes 2} \otimes \mathbb{I}^{\otimes 2})$. In particular, it does not change the operator's spectrum. It follows that, for any state $\tau \in \mathscr{S}$, invariant under $S_{A'A''} \otimes \mathbb{I}_{C'C''}$,

$$\|\rho_0 - \tau\|_1 = \|\rho_0^{T_{AA'}} - \tau^{T_{AA'}}\|_1 = \|\rho_0^{T_{A'A''}} - \tau\|_1. \tag{18}$$

Invoking the triangle inequality, we have that

$$2 = \|\rho_0 - \rho^{T_{A'A''}}\|_1 \leq \|\rho_0 - \tau\|_1 + \|\rho_0^{T_{A'A''}} - \tau\|_1 = 2\|\rho_0 - \tau\|_1, \tag{19}$$

and hence the solution of problem (16) is lower bounded by 1. This bound happens to be tight: it is saturated by taking τ to be the maximally mixed state.

Having solved problem (16), we proceed to prove Theorem 2. We use the same notation as in section VI. Define $\omega := (\mathrm{tr}_{B_1 B_2}\psi) \otimes |0000\rangle\langle 0000|_{A'C'A''C''}$ as Alice and Charlie's state before any measurement by Bob. Consider the state $\rho_\varepsilon := \mathrm{tr}_{AC}(U \otimes V \omega U^T \otimes V^T)$ left on the $A'A''C'C''$ systems after applying over Alice and Charlie's systems AC the real local isometries U, V (see Figure 1) and tracing out AC. As reasoned, such a state must be invariant under transposition of the systems $A'A''$ if Alice, Bob and Charlie's system admits a real quantum representation. Obviously, that will not happen if

$$\|\rho_\varepsilon - \rho_0\|_1 < \min_{\tau \in \mathscr{S}} \|\tau - \rho_0\|_1 = 1. \tag{20}$$

By the triangle inequality, we can bound

$$\|\rho_\varepsilon - \rho_0\|_1 \leq \epsilon_1(\varepsilon) + \epsilon_2(\varepsilon), \tag{21}$$

in terms of $\epsilon_1(\varepsilon) := \|\rho_\varepsilon - \sum_b P(b)\sigma^b\|_1$ and $\epsilon_2(\varepsilon) := \|\sum_b P(b)\sigma^b - \rho_0\|_1$, where $\sigma^b = \mathrm{tr}_{AC}(|\sigma^b\rangle\langle\sigma^b|)$ and $|\sigma^b\rangle$ are the potentially *unnormalized* states

$$|\sigma^b\rangle = \begin{cases} |\phi^+\rangle_{A'C'} \otimes \frac{1}{\sqrt{2}} \left[|\psi^+\rangle_{A''C''} \frac{\mathbb{I}+Z^A}{\sqrt{2}} |\psi^b\rangle_{AC} + |\phi^-\rangle_{A''C''} Y^A X^A \frac{\mathbb{I}+Z^A}{\sqrt{2}} |\psi^b\rangle_{AC} \right] & \text{for } b = 00 = \phi^+ \\ |\psi^+\rangle_{A'C'} \otimes \frac{1}{\sqrt{2}} \left[|\psi^+\rangle_{A''C''} \frac{X^A(\mathbb{I}-Z^A)}{\sqrt{2}} |\psi^b\rangle_{AC} + |\phi^-\rangle_{A''C''} \frac{Y^A(\mathbb{I}-Z^A)}{\sqrt{2}} |\psi^b\rangle_{AC} \right] & \text{for } b = 01 = \psi^+ \\ |\phi^-\rangle_{A'C'} \otimes \frac{1}{\sqrt{2}} \left[|\psi^+\rangle_{A''C''} \frac{\mathbb{I}+Z^A}{\sqrt{2}} |\psi^b\rangle_{AC} + |\phi^-\rangle_{A''C''} Y^A X^A \frac{\mathbb{I}+Z^A}{\sqrt{2}} |\psi^b\rangle_{AC} \right] & \text{for } b = 10 = \phi^- \\ |\psi^-\rangle_{A'C'} \otimes \frac{1}{\sqrt{2}} \left[|\psi^+\rangle_{A''C''} \frac{X^A(\mathbb{I}-Z^A)}{\sqrt{2}} |\psi^b\rangle_{AC} + |\phi^-\rangle_{A''C''} \frac{Y^A(\mathbb{I}-Z^A)}{\sqrt{2}} |\psi^b\rangle_{AC} \right] & \text{for } b = 11 = \psi^-. \end{cases} \tag{22}$$

Thus, if the sum of the upper bounds on $\epsilon_1(\varepsilon)$ and $\epsilon_2(\varepsilon)$ computed in the following sections is smaller than 1, then, by eq. (20), ρ_ε is not reproducible with real quantum states. This leads to a critical error value of $\varepsilon_c = 7.18 \cdot 10^{-5}$.

A. Upper bounds on $\epsilon_1(\varepsilon)$

Lemma 1. *Let $|\psi\rangle$ be a state that, with the measurement operators X^A, Y^A, Z^A for Alice and D^C_{xy}, D^C_{zx}, D^C_{zy}, E^C_{xy}, E^C_{zx}, E^C_{zy} for Charlie, obeys $\langle\psi|\hat{\mathscr{T}}_b|\psi\rangle = 6\sqrt{2} - \varepsilon$, then*

$$\|U \otimes V\,(|\psi\rangle_{AC}|0000\rangle_{A'C'A''C''}) - |\sigma^b\rangle\| \leq (15 + 13\sqrt{2})\varepsilon_1, \tag{23}$$

with $\varepsilon_1 = \sqrt{\sqrt{2}\varepsilon}$ and where $U \otimes V$ denotes the isometry from Figure 1 with $\hat{X}^C$, $\hat{Y}^C$, $\hat{Z}^C$ the regularised versions of $\frac{D^C_{zx} - E^C_{zx}}{\sqrt{2}}$, $\frac{D^C_{zy} - E^C_{zy}}{\sqrt{2}}$ and $\frac{D^C_{zx} + E^C_{zx}}{\sqrt{2}}$ respectively.

Proof. Let

$$\hat{Z}^C := \mathrm{reg}(Z^C) \equiv \mathrm{reg}(Z^C_{zx}), \text{ similarly for } X^C \equiv X^C_{zx} \text{ and } Y^C \equiv Y^C_{zy}, \tag{24}$$

and

$$Z^C_{zx} := \frac{D^C_{zx} + E^C_{zx}}{\sqrt{2}}, \quad X^C_{zx} := \frac{D^C_{zx} - E^C_{zx}}{\sqrt{2}} \tag{25}$$

$$Z^C_{zy} := \frac{D^C_{zy} + E^C_{zy}}{\sqrt{2}}, \quad Y^C_{zy} := \frac{D^C_{zy} - E^C_{zy}}{\sqrt{2}} \tag{26}$$

$$X^C_{xy} := \frac{D^C_{xy} + E^C_{xy}}{\sqrt{2}}, \quad Y^C_{xy} := \frac{D^C_{xy} - E^C_{xy}}{\sqrt{2}} \tag{27}$$

Note that these definitions are independent of b. Then using the sum-of-square (SOS) decomposition

$$\sqrt{2}(6\sqrt{2} - \hat{\mathscr{I}}_b) = \left[(-1)^{b_2} Z^A - \frac{D^C_{zx} + E^C_{zx}}{\sqrt{2}}\right]^2 + \left[(-1)^{b_1} X^A - \frac{D^C_{zx} - E^C_{zx}}{\sqrt{2}}\right]^2 \tag{28}$$

$$+ \left[(-1)^{b_2} Z^A - \frac{D^C_{zy} + E^C_{zy}}{\sqrt{2}}\right]^2 + \left[(-1)^{b_1+b_2} Y^A + \frac{D^C_{zy} - E^C_{zy}}{\sqrt{2}}\right]^2 \tag{29}$$

$$+ \left[(-1)^{b_1} X^A - \frac{D^C_{xy} + E^C_{xy}}{\sqrt{2}}\right]^2 + \left[(-1)^{b_1+b_2} Y^A + \frac{D^C_{xy} - E^C_{xy}}{\sqrt{2}}\right]^2 . \tag{30}$$

we read off the following approximate relations

$$\|((-1)^{b_2} Z^A - Z^C)|\psi\rangle\|, \; \|((-1)^{b_1} X^A - X^C)|\psi\rangle\|, \; \|((-1)^{b_1+b_2} Y^A + Y^C)|\psi\rangle\| \le \varepsilon_1 \, . \tag{31}$$

Using the SOS decomposition

$$\begin{aligned}\sqrt{2}(6\sqrt{2}\mathbb{I} - \hat{\mathscr{I}}_b) = & \left[D^C_{zx} - \frac{(-1)^{b_2} Z^A + (-1)^{b_1} X^A}{\sqrt{2}}\right]^2 + \left[E^C_{zx} - \frac{(-1)^{b_2} Z^A - (-1)^{b_1} X^A}{\sqrt{2}}\right]^2 \\ & + \left[D^C_{zy} - \frac{(-1)^{b_2} Z^A - (-1)^{b_1+b_2} Y^A}{\sqrt{2}}\right]^2 + \left[E^C_{zy} - \frac{(-1)^{b_2} Z^A + (-1)^{b_1+b_2} Y^A}{\sqrt{2}}\right]^2 \\ & + \left[D^C_{xy} - \frac{(-1)^{b_1} X^A - (-1)^{b_1+b_2} Y^A}{\sqrt{2}}\right]^2 + \left[E^C_{xy} - \frac{(-1)^{b_1} X^A + (-1)^{b_1+b_2} Y^A}{\sqrt{2}}\right]^2 \end{aligned} \tag{32}$$

we can prove that

$$\|\{(-1)^{b_2} Z^A, (-1)^{b_1} X^A\}|\psi\rangle\|, \; \|\{(-1)^{b_2} Z^A, (-1)^{b_1+b_2} Y^A\}|\psi\rangle\|, \; \|\{(-1)^{b_1+b_2} Y^A, (-1)^{b_1} X^A\}|\psi\rangle\| \le 2(1+\sqrt{2})\varepsilon_1, \tag{33}$$

i.e., X^A, Y^A, Z^A all anticommute approximately, as follows: Since $(D^C_{zx})^2 = \mathbb{I}$ we get

$$\left[D^C_{zx} + \frac{(-1)^{b_2} Z^A + (-1)^{b_1} X^A}{\sqrt{2}}\right]\left[D^C_{zx} - \frac{(-1)^{b_2} Z^A + (-1)^{b_1} X^A}{\sqrt{2}}\right] = -\frac{\{(-1)^{b_2} Z^A, (-1)^{b_1} X^A\}}{2}. \tag{34}$$

Now apply both sides to $|\psi\rangle$ and take the norm; we get the desired inequality after noticing that the operator norm of the first square bracket is bounded by $1+\sqrt{2}$ (by triangle inequality and D^C_{zx} being unitary). Finally, the regularized operators are also close to the unregularized counterparts, e.g.

$$\|(\hat{Z}^C - Z^C)|\psi\rangle\| = \|(\mathbb{I} - (\hat{Z}^C)^\dagger Z^C)|\psi\rangle\| = \|(\mathbb{I} - |Z^C|)|\psi\rangle\| = \|(\mathbb{I} - |Z^A Z^C|)|\psi\rangle\| \le \|(\mathbb{I} - Z^A Z^C)|\psi\rangle\| \le \varepsilon_1. \tag{35}$$

Now we apply the isometry $U \otimes V$ defined in Figure 1. The cancellation happens exactly as in the ideal case incurring a small loss measured in vector norm because relations are only approximate. After $U' \otimes V'$ (see Figure 1), the unknown state is close to a (potentially unnormalized) vector in a specific form

$$\|U' \otimes V'(|\psi\rangle_{AC}|00\rangle_{A'C'}) - \mathscr{O}(b)|\psi\rangle_{AC}|b\rangle_{A'C'}\| \le (5+\sqrt{2})\varepsilon_1 \tag{36}$$

where

$$\mathscr{O}(b) = \begin{cases} \frac{\mathbb{I}+Z^A}{\sqrt{2}} & \text{if } b_2 = 0 \\ \frac{X^A(\mathbb{I}-Z^A)}{\sqrt{2}} & \text{if } b_2 = 1 \end{cases} \tag{37}$$

To get this result, we compute the effect of the different steps of the isometry on the initial vector and make use of the relations (31), (33), (35) in order to simplify the final expression, all the while keeping track of the error incurred to at each stage. That is, we first apply the Hadamard gates, and then control Z^A and $\hat{Z}^C$ gates, which return a state as written in the first line of Step 1 below. Since so far we did not use any of the approximate relations, the error incurred to in this step is 0, as written on the left of the state in Step 1. Next, we use the approximate relation (31) to convert $Z^A\hat{Z}^C|\psi\rangle$ to $(-1)^{b_2}Z^AZ^A|\psi\rangle = (-1)^{b_2}|\psi\rangle$, and similarly $\hat{Z}^C|\psi\rangle$ to $\hat{Z}^A|\psi\rangle$, incurring into an error $2\varepsilon_1$, written on the left. Continuing this way through the circuit, the intermediate expressions, together with their bounds, are the following:

$$\begin{aligned}
\text{Step 1:}\qquad & 0\varepsilon_1 : \frac{1}{2}\Big[|00\rangle + |11\rangle Z^A\hat{Z}^C + |01\rangle\hat{Z}^C + |10\rangle Z^A\Big]|\psi\rangle \\
& 2\varepsilon_1 : \frac{1}{2}\left[(|00\rangle + (-1)^{b_2}|11\rangle)\mathbb{I} + ((-1)^{b_2}|01\rangle + |10\rangle)Z^A\right]|\psi\rangle \\
\text{Step 2:}\qquad & 0\varepsilon_1 : \frac{1}{4}\Big[|00\rangle(1+(-1)^{b_2})(\mathbb{I}+Z^A) + |11\rangle(1+(-1)^{b_2})X^A\hat{X}^C(\mathbb{I}-Z^A) + \\
& \qquad |01\rangle(1-(-1)^{b_2})\hat{X}^C(\mathbb{I}+Z^A) + |10\rangle(1-(-1)^{b_2})X^A(\mathbb{I}-Z^A))\Big]|\psi\rangle \\
& (3+\sqrt{2})\varepsilon_1 : \frac{1}{4}\left[(1+(-1)^{b_2})(|00\rangle + (-1)^{b_1}|11\rangle)(\mathbb{I}+Z^A) + (1-(-1)^{b_2})((-1)^{b_1}|01\rangle + |10\rangle)X^A(\mathbb{I}-Z^A))\right]|\psi\rangle
\end{aligned}$$

Note that in the last approximation, the two paths $b_2 = 0$ and $b_2 = 1$ have the same upper bound. Also, we use the convention that $|\psi^-\rangle = (|10\rangle - |01\rangle)/\sqrt{2}$, which has the same density matrix as the usual convention.

The rest of the circuit does not involve $A'C'$ so we can safely ignore them. After the remaining local unitaries $U'' \otimes V''$ (see Figure 1),

$$\|U'' \otimes V''\left(\mathscr{O}(b)|\psi\rangle_{AC}|00\rangle_{A''C''}\right) - |\tau^b\rangle\| \le (10+12\sqrt{2})\varepsilon_1, \tag{38}$$

where

$$|\tau^b\rangle = \begin{cases} \frac{1}{\sqrt{2}}\left[|\psi^+\rangle\frac{\mathbb{I}+Z^A}{\sqrt{2}}|\psi\rangle + |\phi^-\rangle Y^AX^A\frac{\mathbb{I}+Z^A}{\sqrt{2}}|\psi\rangle\right] & \text{for } b_2 = 0 \\ \frac{1}{\sqrt{2}}\left[|\psi^+\rangle\frac{X^A(\mathbb{I}-Z^A)}{\sqrt{2}}|\psi\rangle + |\phi^-\rangle\frac{Y^A(\mathbb{I}-Z^A)}{\sqrt{2}}|\psi\rangle\right] & \text{for } b_2 = 1 \end{cases} \tag{39}$$

The intermediate steps are

$$\begin{aligned}
\text{Step 3:}\qquad & 0\varepsilon_1 : \frac{1}{2}\Big[|00\rangle\mathscr{O}(b) + |11\rangle Y^AX^A\hat{Y}^C\hat{X}^C\mathscr{O}(b) + |01\rangle\hat{Y}^C\hat{X}^C\mathscr{O}(b) + |10\rangle Y^AX^A\mathscr{O}(b)\Big]|\psi\rangle \\
& (8+10\sqrt{2})\varepsilon_1 : \frac{1}{2}\left[|00\rangle\mathscr{O}(b) + |11\rangle Y^AX^AY^AX^A\mathscr{O}(b) + |01\rangle Y^AX^A\mathscr{O}(b) + |10\rangle Y^AX^A\mathscr{O}(b)\right]|\psi\rangle \\
& (2+2\sqrt{2})\varepsilon_1 : \frac{1}{2}\left[(|00\rangle - |11\rangle)\mathscr{O}(b) + (|01\rangle + |10\rangle)Y^AX^A\mathscr{O}(b)\right]|\psi\rangle \\
\text{Step 4:}\qquad & 0\varepsilon_1 : \frac{1}{2}\left[(|01\rangle + |10\rangle)\mathscr{O}(b) + (|00\rangle - |11\rangle)Y^AX^A\mathscr{O}(b)\right]|\psi\rangle
\end{aligned}$$

where we have taken the larger bound among the two $b_2 = 1$ and $b_2 = 0$ cases to get

$$\|(\hat{Y}^C\hat{X}^C\mathscr{O}(b) - Y^AX^A\mathscr{O}(b))|\psi\rangle\| \le (20+8\sqrt{2})\varepsilon_1/\sqrt{2} \text{ and} \tag{40}$$

$$\|(Y^AX^AY^AX^A\mathscr{O}(b) + \mathscr{O}(b))|\psi\rangle\| \le (8+4\sqrt{2})\varepsilon_1/\sqrt{2}\,. \tag{41}$$

By a series of triangle inequalities going through all the intermediate expressions, we get the Lemma. □

Lemma 2. *Let* $|\sigma^b\rangle$ *be any state defined in* (22) *and let* $\varepsilon_1 = \sqrt{\sqrt{2}\varepsilon}$, *then*

$$1 - (3+\sqrt{2})\varepsilon_1 \le \||\sigma^b\rangle\|^2 \le 1 + (3+\sqrt{2})\varepsilon_1 \tag{42}$$

Proof. With $\||\sigma^b\rangle\|^2 = \mathrm{tr}|\sigma^b\rangle\langle\sigma^b|$ then

$$|1 - \mathrm{tr}|\sigma^b\rangle\langle\sigma^b|| = |\langle\psi^b|Z^A|\psi^b\rangle|. \tag{43}$$

Now consider, as in [24],

$$\begin{aligned}|\langle\psi^b|Z^A|\psi^b\rangle+\langle\psi^b|Z^AX^A\hat{X}^C|\psi^b\rangle| &= |\langle\psi^b|Z^A|\psi^b\rangle+\langle\psi^b|\hat{X}^CX^AZ^A|\psi^b\rangle|\\ &= |\langle\psi^b|(\hat{X}^CZ^A\hat{X}^C+\hat{X}^CX^AZ^A)|\psi^b\rangle|\\ &\leq \|\hat{X}^C|\psi^b\rangle\|\|(Z^A\hat{X}^C+X^AZ^A)|\psi^b\rangle\| \leq (4+2\sqrt{2})\varepsilon_1.\\ |\langle\psi^b|Z^A|\psi^b\rangle-\langle\psi^b|Z^AX^A\hat{X}^C|\psi^b\rangle| &\leq \|(Z^A(\mathbb{I}-X^A\hat{X}^C)|\psi^b\rangle\|\\ &\leq \|Z^A(\hat{X}^C-X^A)|\psi^b\rangle\| \leq 2\varepsilon_1,\end{aligned}$$

where we used the Cauchy-Schwarz inequality as well as

$$\|(Z^A\hat{X}^C+X^AZ^A)|\psi^b\rangle\| \leq \|(Z^A\hat{X}^C-Z^AX^A)|\psi^b\rangle\|+\|\{X^A,Z^A\}|\psi^b\rangle\| \leq 2\varepsilon_1+2(1+\sqrt{2})\varepsilon_1.$$

Therefore, we find that

$$|\langle\psi^b|Z^A|\psi^b\rangle| \leq (3+\sqrt{2})\varepsilon_1, \tag{44}$$

which combined with (43) implies (42). □

Using these Lemmas, we can obtain the final bound for $\epsilon_1(\varepsilon)$. Firstly, we have

$$\epsilon_1(\varepsilon) \leq \sum_b P(b)\||\rho_\varepsilon^b\rangle\langle\rho_\varepsilon^b|-|\sigma^b\rangle\langle\sigma^b|\|_1 \leq 2\sum_b P(b)\sqrt{\frac{(1+\||\sigma^b\rangle\|^2)^2}{4}-|\langle\rho_\varepsilon^b|\sigma^b\rangle|^2}, \tag{45}$$

where the first equality comes from $\rho_\varepsilon = \sum_b P(b)\rho_\varepsilon^b$ for ρ_ε^b the $A'C'A''C''$ marginal of the state $|\rho_\varepsilon^b\rangle = U\otimes V(|\psi^b\rangle_{AC}|0000\rangle^{A'C'A''C''})$. The last inequality follows by adapting an argument from [25] to non-normalised states. Namely, since $|\rho_\varepsilon^b\rangle\langle\rho_\varepsilon^b|-|\sigma^b\rangle\langle\sigma^b|$ has rank at most two,

$$\begin{aligned}\lambda_1+\lambda_2 &= \operatorname{tr}(|\rho_\varepsilon^b\rangle\langle\rho_\varepsilon^b|-|\sigma^b\rangle\langle\sigma^b|) &&= 1-\||\sigma^b\rangle\|^2\\ \lambda_1^2+\lambda_2^2 &= \operatorname{tr}((|\rho_\varepsilon^b\rangle\langle\rho_\varepsilon^b|-|\sigma^b\rangle\langle\sigma^b|)^2) &&= 1+\||\sigma^b\rangle\|^4-2|\langle\rho_\varepsilon^b|\sigma^b\rangle|^2,\end{aligned}$$

where λ_1 and λ_2 are the two possibly non-zero eigenvalues of $|\rho_\varepsilon^b\rangle\langle\rho_\varepsilon^b|-|\sigma^b\rangle\langle\sigma^b|$. Solving explicitly for λ_1 and λ_2 in the previous system of equations shows that

$$|\lambda_1|+|\lambda_2| = 2\sqrt{\frac{(1+\||\sigma^b\rangle\|^2)^2}{4}-|\langle\rho_\varepsilon^b|\sigma^b\rangle|^2}.$$

Furthermore,

$$\langle\rho_\varepsilon^b|\sigma^b\rangle = \frac{1+\||\sigma^b\rangle\|^2-\||\rho_\varepsilon^b\rangle-|\sigma^b\rangle\|^2}{2}$$

which implies, by Lemma 1 and Lemma 2, that for small $\varepsilon>0$,

$$\epsilon_1(\varepsilon) \leq 2\sqrt{\frac{[1+(1+(3+\sqrt{2})\varepsilon_1)]^2}{4}-\left(1-\frac{(3+\sqrt{2})\varepsilon_1+\varepsilon_2^2}{2}\right)^2} \text{ with } \varepsilon_2 := (15+13\sqrt{2})\varepsilon_1. \tag{46}$$

B. Upper bounds on $\epsilon_2(\varepsilon)$

To bound $\epsilon_2(\varepsilon) = \|\sum_b P(b)\sigma^b-\rho_0\|_1$, let us first separate the expression in the norm as

$$\|\sigma^b-\rho_0^b\|_1 \leq |\hat{\mu}(b)|\||b\rangle\langle b|\otimes(|\phi^-\rangle\langle\psi^+|+|\psi^+\rangle\langle\phi^-|)\|_1+|\mu(b)|\||b\rangle\langle b|\otimes(|\phi^-\rangle\langle\phi^-|+|\psi^+\rangle\langle\psi^+|)\|_1,$$

where the coefficients simplify to $|\mu(b)| = \frac{1}{2}|\langle\psi^b|Z^A|\psi^b\rangle|$ and

$$|\hat{\mu}(b)| = \begin{cases} \frac{1}{4}|\langle\psi^b|(\mathbb{I}+Z^A)X^AY^A(\mathbb{I}+Z^A)|\psi^b\rangle| & b_2=0 \\ \frac{1}{4}|\langle\psi^b|(\mathbb{I}-Z^A)X^AY^A(\mathbb{I}-Z^A)|\psi^b\rangle| & b_2=1. \end{cases}$$

To bound $|\hat{\mu}(b)|$, let us consider

$$\begin{aligned} \frac{1}{4}|\langle\psi^b|(\mathbb{I}+(-1)^{b_2}Z^A)\{X^A,Y^A\}(\mathbb{I}+(-1)^{b_2}Z^A)|\psi^b\rangle| &\leq \frac{1}{4}\|(\mathbb{I}+(-1)^{b_2}Z^A)|\psi^b\rangle\|\|\{X^A,Y^A\}(\mathbb{I}+(-1)^{b_2}Z^A)|\psi^b\rangle\| \\ &\leq \frac{1}{2}\|\{X^A,Y^A\}(\mathbb{I}+(-1)^{b_2}Z^A)|\psi^b\rangle\| \\ &\leq \frac{1}{2}(\|\{X^A,Y^A\}|\psi^b\rangle\| \\ &+\|\hat{Z}^C\{X^A,Y^A\}|\psi^b\rangle\|+\|\{X^A,Y^A\}((-1)^{b_2}Z^A-\hat{Z}^C)|\psi^b\rangle\|) \\ &\leq (4+2\sqrt{2})\varepsilon_1. \end{aligned} \tag{47}$$

Therefore, using (47) and (44), we obtain

$$\|\sigma^b-\rho_0^b\|_1 \leq 2(|\hat{\mu}(b)|+|\mu(b)|) \leq (4+2\sqrt{2})\varepsilon_1+(3+\sqrt{2})\varepsilon_1 = (7+3\sqrt{2})\varepsilon_1.$$

Since $|P(b)-\frac{1}{4}| \leq \varepsilon$, we obtain

$$\epsilon_2(\varepsilon) \leq \sum_b \frac{1}{4}\|\sigma^b-\rho_0^b\|_1 + \sum_b |P(b)-\frac{1}{4}|\|\sigma^b\|_1 \leq (7+3\sqrt{2})\varepsilon_1+4(1+(3+\sqrt{2})\varepsilon_1)\epsilon \tag{48}$$

VIII. PROOF OF THEOREM 3

Our first step is to obtain a tractable necessary condition for a distribution P to admit a real quantum realization. To this aim, we invoke the essentials of non-commutative polynomial optimization [18].

Let P admit a real quantum representation of the form (5), and think of an abstract unital $\star$-algebra $\mathscr{A}$ with generators $A_{1|1}, A_{1|2}, A_{1|3}$, together with the relations $A_{1|x} = A_{1|x}^\dagger = (A_{1|x})^2$, for $x = 1,2,3$. We consider the natural isomorphism π_A that maps any element of $\mathscr{A}$ to the algebra generated by the operators $\{\tilde{A}_{1|x} : x = 1,2,3\}$. That is, $A_{1|x}$ is an element of $\mathscr{A}$, while $\pi_A(A_{1|x}) = \tilde{A}_{1|x}$ represents Alice's physical measurement operator for setting x and outcome $a = 1$. We call $\mathcal{A} \subset \mathscr{A}$ the set of all monomials of degree n_A or lower of $A_{1|1}, A_{1|2}, A_{1|3} \in \mathscr{A}$ including the identity, for some fixed natural number n_A. Similarly, we define an abstract unital $\star$-algebra $\mathscr{C}$ with projection generators $C_{1|1}, ..., C_{1|6}$ to model Charlie's measurements, with the natural isomorphism π_C. We denote by $\mathcal{C} \subset \mathscr{C}$ the set of all monomials of degree n_C or lower of $C_{1|1}, ..., C_{1|6}$, including the identity for some fixed n_C.

Following Moroder *et al.*'s interpretation [26] of the Navascués-Pironio-Acín (NPA) hierarchy [27, 28], to each monomial $\alpha \in \mathscr{A}$ we associate a normalized ket $|\alpha\rangle$ such that $\langle\alpha'|\alpha\rangle = \delta_{\alpha,\alpha'}$, for all $\alpha, \alpha' \in \mathscr{A}$. Likewise, we associate an orthonormal basis to the set $\mathscr{C}$. Next, we define the completely positive local maps Ω_A, Ω_C through the relations:

$$\begin{aligned} \Omega_A(\eta) &= \sum_{\alpha,\alpha' \in \mathcal{A}} \mathrm{tr}\left((\pi_A(\alpha)^\dagger \eta \pi_A(\alpha')\right)|\alpha\rangle\langle\alpha'| \\ \Omega_C(\eta) &= \sum_{\gamma,\gamma' \in \mathcal{C}} \mathrm{tr}\left(\pi_C(\gamma)^\dagger \eta \pi_C(\gamma')\right)|\gamma\rangle\langle\gamma'|. \end{aligned} \tag{49}$$

Ω_A (Ω_C) thus maps states in Alice's (Charlie's) untrusted system A (C) to a non-normalized state with support in $H_A = \mathrm{span}\{|\alpha\rangle : \alpha \in \mathcal{A}\}$ ($H_C = \mathrm{span}\{|\gamma\rangle : \gamma \in \mathcal{C}\}$).

For $\lambda \in \Lambda$, define the state $\tilde{\omega}^b(\lambda) \equiv \mathrm{tr}_{B_1B_2}\left\{(\tilde{\sigma}^\lambda_{AB_1} \otimes \tilde{\sigma}^\lambda_{B_2C})(\mathbb{I}_A \otimes \tilde{B}_b \otimes \mathbb{I}_C)\right\}$ and consider the $|\mathcal{A}||\mathcal{C}| \times |\mathcal{A}||\mathcal{C}|$ matrix

$$\Gamma^b \equiv \sum_\lambda P(\lambda)(\Omega_A \otimes \Omega_C)(\tilde{\omega}^b(\lambda)). \tag{50}$$

Since Ω_A, Ω_C are completely positive, this matrix must be positive semidefinite. Moreover, some of its entries are related. Indeed, let the monomials $\alpha_1, ..., \alpha_4 \in \mathcal{A}$, $\gamma_1, ..., \gamma_4 \in \mathcal{C}$ be such that $\alpha_2\alpha_1^\dagger = \alpha_4\alpha_3^\dagger =: \alpha$, $\gamma_2\gamma_1^\dagger = \gamma_4\gamma_3^\dagger =: \gamma$. Then it holds that

$$\langle\alpha_1|\langle\gamma_1|\Gamma^b|\alpha_2\rangle|\gamma_2\rangle = \langle\alpha_3|\langle\gamma_3|\Gamma^b|\alpha_4\rangle|\gamma_4\rangle = \sum_\lambda P(\lambda)\text{tr}\{\tilde{\omega}^b(\lambda)(\pi_A(\alpha)\otimes\pi_C(\gamma))\}. \tag{51}$$

This allows us to write Γ^b as

$$\Gamma^b = \sum_{\alpha\in\mathcal{A}\cdot\mathcal{A},\gamma\in\mathcal{C}\cdot\mathcal{C}} d^b_{\alpha,\gamma} M^\alpha \otimes N^\gamma, \tag{52}$$

where $\{d^b_{\alpha,\gamma} : \alpha, \gamma\}$ are real coefficients, and, for any $a, a' \in \mathcal{A}, c, c' \in \mathcal{C}$, the corresponding entries of M^α, N^γ are given by

$$M^\alpha_{a,a'} = \delta_{\alpha,a'a^\dagger}, N^\gamma_{c,c'} = \delta_{\gamma,c'c^\dagger}. \tag{53}$$

Also, notice that some of the coefficients $d^b_{\alpha,\gamma}$ follow from the experimental data $P(a,b,c|x,z)$. Namely,

$$d^b_{\mathbb{I},\mathbb{I}} = P(b), d^b_{A_{1|x},\mathbb{I}} = P_{AB}(1,b|x), d^b_{\mathbb{I},C_{1|z}} = P_{BC}(b,1|z), d^b_{A_{1|x},C_{1|z}} = P(1,b,1|x,z). \tag{54}$$

In the terminology of [18], Γ^b is a non-normalized *moment matrix* for the distribution $P(a,c|x,z,b)$, with norm $d^b_{\mathbb{I},\mathbb{I}} = P(b)$.

Consider now the matrix $\Gamma \equiv \sum_b \Gamma^b$. From the definition of $\tilde{\omega}^b(\lambda)$, we have that, for any $\lambda \in \Lambda$, $\sum_b \tilde{\omega}^b(\lambda) = \tilde{\sigma}^\lambda_A \otimes \tilde{\sigma}^\lambda_C$. It follows that

$$\Gamma = \sum_\lambda P(\lambda)\Omega_A(\tilde{\sigma}^\lambda_A) \otimes \Omega_C(\tilde{\sigma}^\lambda_C). \tag{55}$$

Since Ω_A, Ω_C are real completely positive maps, we have that Γ is a real separable operator, i.e., a conic combination of real product quantum states. In particular, it must be that Γ equals its partial transpose, $\Gamma^{T_A} = \Gamma$ [23].

To summarize: if P admits a real quantum representation in the SWAP scenario, then there must exist real coefficients $d^b_{\alpha,\gamma}$ such that eq. (54) holds, the matrices Γ^b defined through eq. (52) are positive semidefinite and the matrix $\sum_b \Gamma^b$ is its own partial transpose. Consider then the following optimization problem:

$$\begin{aligned}
\max_{d,P} \quad & \mathscr{T}(P), \\
\text{such that} \quad & \Gamma^b = \sum_{\alpha\in\mathcal{A}\cdot\mathcal{A},\gamma\in\mathcal{C}\cdot\mathcal{C}} d^b_{\alpha,\gamma} M^\alpha \otimes N^\gamma \geq 0, \text{ for } b = 1, ..., 4, \\
& (\sum_b \Gamma^b)^{T_A} = \sum_b \Gamma^b, \\
& d^b_{\mathbb{I},\mathbb{I}} = P(b), d^b_{A_{1|x},\mathbb{I}} = P_{AB}(1,b|x), d^b_{\mathbb{I},C_{1|z}} = P_{BC}(b,1|z), d^b_{A_{1|x},C_{1|z}} = P(1,b,1|x,z), \\
& P(a,b,c|x,z) \geq 0, \sum_a P(a,b,c|x,z) = P_{BC}(b,c|z), \sum_c P(a,b,c|x,z) = P_{AB}(a,b|x), \\
& \sum_a P_{AB}(a,b|x) = \sum_c P_{BC}(b,c|z) = P(b), \sum_b P(b) = 1,
\end{aligned} \tag{56}$$

where the conditions on $P(a,b,c|x,z)$ in the last two lines enforce that P corresponds to a non-signalling, normalized tripartite distribution.

The above is a semidefinite program (SDP) [19], and, as long as the matrices M^α, N^γ are not very large, one can find the solution in a normal desktop. Since the constraints enforce a relaxation of the requirement that P admit a representation in the SWAP scenario, it follows that the solution of this problem is an upper bound on the maximum value of $\mathscr{T}(P)$ for P achievable through real quantum systems in the considered causal structure.

Taking $n_A = n_C = 2$, and using the SDP solver MOSEK [20] within the optimization package YALMIP [21], we arrive at Theorem 3.

IX. EXPERIMENTAL CONSIDERATIONS

In view of Theorem 3, there exists a considerable gap between the predictions of real and complex quantum theory in the entanglement swapping scenario. Does that mean that an actual experimental refutation of real quantum theory is within reach? Let us briefly consider how an experimental realization of the quantum experiment depicted in Figure 2 of the main text (up) would go.

First, a general observation: even if our proposed experimental setup mimics that of Figure 2 of the main text, in principle, there could exist prior quantum correlations between the two preparation devices, or a quantum state shared by the three parties as a result of a past quantum interaction (say, in the last round of experiments). In either predicament, our previous bounds on the input-output statistics for real quantum system would not be valid, and any claims of refutation of real quantum physics would be unfounded. These two possibilities, though, rely on the presence of hidden quantum memories within the experimental equipment. Hence, if we posit a time-scale beyond which the devices' quantum memories degrade into classical information, we can discard such contingencies just by spacing out the experimental rounds sufficiently (note that prior classical correlations between the parties can be absorbed into the definition of λ).

There is another loophole, namely, the possible existence of hidden state sources that distribute general tripartite entangled states at each experimental round. This would similarly compromise the conclusions of the experiment, and cannot be ruled out by appealing to decoherence or space-like separation. In order to refute real quantum physics, we are thus compelled to accept some plausible, yet unverifiable, assumptions about the form of the quantum states distributed to the three parties. In the following, we therefore *postulate* that Figure 2 of the main text (down) accurately captures the causal scenario encountered by the three parties at each experimental round. Crucially, we allow the states $\tilde{\sigma}^{\lambda}_{AB_1}, \tilde{\sigma}^{\lambda}_{B_2C}$ and the distribution $P(\lambda)$ to depend in arbitrary ways on the past history h of measurement settings and outcomes observed by the three parties in the course of the experiment. That is, we allow the real quantum physicist to adapt its states and measurement operators at each round to make us believe that it holds complex quantum resources.

Under the above adversarial conditions, the inequality $\mathscr{T} \leq 7.6605$ nonetheless holds at every experimental round. Therefore, one can use the techniques in [29] and [30] to devise an n-round experiment that, if successful, disproves the hypothesis of real quantum physics with high statistical confidence.

Of course, in order to get there, one first needs to realize a quantum entanglement swapping experiment with $\mathscr{T} > 7.6605$. We next discuss the technical feasibility of this goal. We assume that $\bar{\sigma}_{AB}, \bar{\sigma}_{BC}$ are distributed via photon sources, and that photon polarization measurements are almost perfect. We then face two experimental problems: making sure that the photons reach their destination more or less unperturbed and conducting the Bell measurement. With regards to the first problem, we model the interaction between the photons and the environment through white noise. That is, rather than being distributed two maximally entangled states, Alice and Bob and Bob and Charlie respectively receive an independent copy of the state $v\Phi^+ + \frac{1-v}{4}\mathbb{1}$. Under ideal Bell measurements, the violation of the inequality $\mathscr{T} \leq 7.6605$ thus requires each photon source to have a visibility v of at least $v = \sqrt{\frac{7.6605}{6\sqrt{2}}} \approx 0.95$, a value realistic with present technology. With regards to the second problem, deterministic Bell-state measurements with photons are indeed complicated [31], but they can be carried out with arbitrary precision, provided that sufficiently many single-photon sources are available [32]. All in all, the experimental requirements to violate (8) are demanding, but within reach.

---

[1] R. Haag, *Local Quantum Physics: Fields, Particles, Algebras*, 1st ed. (Springer Publishing Company, Incorporated, 2012).

[2] R. Werner, Local preparability of states and the split property in quantum field theory, Lett. Math. Phys. **13**, 325 (1987).

[3] D. Buchholz, Product states for local algebras, Commun. Math. Phys. **36**, 287–304 (1974).

[4] S. J. Summers, Normal product states for fermions and twisted duality for ccr- and car-type algebras with application to the yukawa2 quantum field model, Commun. Math. Phys. **86**, 111–141 (1982).

[5] D. Buchholz and P. Jacobi, On the nuclearity condition for massless fields, Lett. Math. Phys. **13**, 313–323 (1987).

[6] E. C. Stueckelberg, Quantum theory in real hilbert space, Helv. Phys. Acta **33**, 458 (1960).

[7] A. Aleksandrova, V. Borish, and W. K. Wootters, Real-vector-space quantum theory with a universal quantum bit, Physical Review A **87**, 10.1103/physreva.87.052106 (2013).

[8] D. Bohm, A suggested interpretation of the quantum theory in terms of "hidden" variables. i, Phys. Rev. **85**, 166 (1952).

[9] W. K. Wootters, Complexity, entropy, and the physics of information (Addison-Wesley, 1990) Chap. Local Accessibility of Quantum States.

[10] C. M. Caves, C. A. Fuchs, and R. Schack, Unknown quantum states: The quantum de finetti representation, Journal of Mathematical Physics **43**, 4537 (2002), https://doi.org/10.1063/1.1494475.

[11] L. Hardy, Quantum theory from five reasonable axioms (2001).

[12] G. Chiribella, G. M. D'Ariano, and P. Perinotti, Informational derivation of quantum theory, Physical Review A **84**, 10.1103/physreva.84.012311 (2011).
[13] G. M. D'Ariano, M. Erba, and P. Perinotti, Classicality without local discriminability: Decoupling entanglement and complementarity, Phys. Rev. A **102**, 052216 (2020).
[14] V. Moretti and M. Oppio, Quantum theory in real hilbert space: How the complex hilbert space structure emerges from poincaré symmetry, Reviews in Mathematical Physics **29**, 1750021 (2017).
[15] J. C. Baez, Division algebras and quantum theory, Foundations of Physics **42**, 819–855 (2011).
[16] F. J. Dyson, The threefold way. algebraic structure of symmetry groups and ensembles in quantum mechanics, Journal of Mathematical Physics **3**, 1199 (1962), https://doi.org/10.1063/1.1703863.
[17] M. F. Pusey, J. Barrett, and T. Rudolph, On the reality of the quantum state, Nature Physics **8**, 475 (2012).
[18] S. Pironio, M. Navascués, and A. Acín, Convergent relaxations of polynomial optimization problems with noncommuting variables, SIAM Journal on Optimization **20**, 2157 (2010), https://doi.org/10.1137/090760155.
[19] L. Vandenberghe and S. Boyd, Semidefinite programming, SIAM Review **38**, 49 (1996).
[20] L. Vandenberghe and S. Boyd, *The MOSEK optimization toolbox for MATLAB manual. Version 7.0 (Revision 140).* (MOSEK ApS, Denmark.).
[21] J. Löfberg, Yalmip : A toolbox for modeling and optimization in matlab, in *Proceedings of the CACSD Conference* (Taipei, Taiwan, 2004).
[22] J. Bowles, I. Šupić, D. Cavalcanti, and A. Acín, Self-testing of pauli observables for device-independent entanglement certification, Phys. Rev. A **98**, 042336 (2018).
[23] C. M. Caves, C. A. Fuchs, and P. Rungta, Entanglement of formation of an arbitrary state of two rebits, Foundations of Physics Letters **14**, 199–212 (2001).
[24] M. McKague, T. H. Yang, and V. Scarani, Robust self-testing of the singlet, Journal of Physics A: Mathematical and Theoretical **45**, 455304 (2012).
[25] J. Watrous, *The theory of quantum information* (Cambridge University Press, 2018).
[26] T. Moroder, J.-D. Bancal, Y.-C. Liang, M. Hofmann, and O. Gühne, Device-independent entanglement quantification and related applications, Phys. Rev. Lett. **111**, 030501 (2013).
[27] M. Navascués, S. Pironio, and A. Acín, Bounding the set of quantum correlations, Phys. Rev. Lett. **98**, 010401 (2007).
[28] M. Navascués, S. Pironio, and A. Acín, A convergent hierarchy of semidefinite programs characterizing the set of quantum correlations, New J. Phys. **10**, 073013 (2008).
[29] D. Elkouss and S. Wehner, (nearly) optimal p-values for all bell inequalities, npj Quantum Information **2**, 16026 (2016).
[30] M. Araújo, F. Hirsch, and M. T. Quintino, Bell nonlocality with a single shot, Quantum **4**, 353 (2020).
[31] J. Calsamiglia and N. Lütkenhaus, Maximum efficiency of a linear-optical bell-state analyzer, Applied Physics B **72**, 67–71 (2001).
[32] W. P. Grice, Arbitrarily complete bell-state measurement using only linear optical elements, Phys. Rev. A **84**, 042331 (2011).
